# Feasibility and guidelines for the use of an injectable fiducial marker (BioXmark ^®^) to improve target delineation in preclinical radiotherapy studies using mouse models.

**DOI:** 10.12688/f1000research.130883.1

**Published:** 2023-05-22

**Authors:** Kathryn Brown, Mihaela Ghita, Kevin M Prise, Karl T Butterworth

**Affiliations:** 1 Patrick G. Johnston Centre for Cancer Research, Queen's University Belfast, Belfast, BT9 7AE, UK

**Keywords:** Fiducial marker, practicable guidelines, preclinical models, image-guidance, preclinical radiotherapy

## Abstract

Background: Preclinical models of radiotherapy (RT) response are vital for the continued success and evolution of RT in the treatment of cancer. The irradiation of tissues in mouse models necessitates high levels of precision and accuracy to recapitulate clinical exposures and limit adverse effects on animal welfare. This requirement has been met by technological advances in preclinical RT platforms established over the past decade. Small animal RT systems use onboard computed tomography (CT) imaging to delineate target volumes and have significantly refined radiobiology experiments with major 3Rs impacts. However, the CT imaging is limited by the differential attenuation of tissues resulting in poor contrast in soft tissues. Clinically, radio-opaque fiducial markers (FMs) are used to establish anatomical reference points during treatment planning to ensure accuracy beam targeting, this approach is yet to translate back preclinical models. Methods: We report on the use of a novel liquid FM BioXmark ^^®^^ developed by Nanovi A/S (Kongens Lyngby, Denmark) that can be used to improve the visualisation of soft tissue targets during beam targeting and minimise dose to surrounding organs at risk. We present descriptive protocols and methods for the use of BioXmark ^^®^^ in experimental male and female C57BL/6J mouse models. Results: These guidelines outline the optimum needle size for uptake (18-gauge) and injection (25- or 26-gauge) of BioXmark ^^®^^ for use in mouse models along with recommended injection volumes (10-20 µl) for visualisation on preclinical cone beam CT (CBCT) scans. Injection techniques include subcutaneous, intraperitoneal, intra-tumoral and prostate injections. Conclusions: The use of BioXmark ^^®^^ can help to standardise targeting methods, improve alignment in preclinical image-guided RT and significantly improve the welfare of experimental animals with the reduction of normal tissue exposure to RT.

## Introduction

Radiotherapy (RT) remains a critical component of multidisciplinary cancer care. Progress in RT has been driven by advances in RT and imaging technologies with parallel increases in our understanding of RT response at the cell, tissue and whole organism levels. RT relies on image guidance to achieve high levels of precision and accuracy during treatment, most often using computed tomography (CT) to maximise the competing probabilities of tumour control (TCP) and normal tissue complication probability (NTCP).

During RT, fiducial markers (FMs) are commonly used for daily alignment, tumour tracking and to improve patient positioning for treatment. ^1^^–^^3^ FMs can enhance the differentiation of tumour and normal tissue margins in low contrast tissues ^4^ and their use has significantly improved the accuracy of different RT techniques: intensity modulated RT (IMRT), image-guided RT, volumetric modulated arc therapy (VMAT) and hypo-fractionated stereotactic treatments. ^5^

Irradiation of tumours and tissues in mouse models requires high levels of precision and accuracy to recapitulate clinical scenarios and limit adverse effects on animal welfare. Commonly, preclinical RT set-ups are untargeted leading to mice presenting with signs of radiation injury and toxicity making studies short-lived hindering further development. ^6^^–^^8^ However, implementation of small animal image-guided radiotherapy platforms in research has been a huge refinement, combining volumetric imaging modalities with treatment planning and delivery. ^9^^–^^11^ These platforms have advanced previous wide field and untargeted RT set-ups, yet the localisation of small, low contrast tissue targets continues to be challenging. ^12^ This has prevented the development of sophisticated and cutting-edge RT protocols, such as hypo-fractionation (delivering a higher dose of radiation per session in comparison to conventional RT therefore reducing the overall number of treatment sessions), due to limitations in visualisation of some soft tissue targets. ^13^ To overcome targeting errors, lack of standardisation in treatment delivery and improve animal welfare in preclinical models of RT without the need for expensive updated platforms we propose the reverse translation of FMs. ^12^

FMs are typically composed of high-Z number materials to ensure differentiation from internal structures. ^14^ Solid FMs, such as gold, are not suitable for preclinical RT as they result in streaking imaging artefacts on CBCT scans, decreased accuracy for dose calculations and alter dose perturbations. ^15^^–^^19^ In addition, the surgical procedure to insert solid FMs puts mice under high levels of stress and decreases animal welfare. ^5^^,^^20^

Liquid FMs, such as BioXmark ^®^, offer refinements over solid fiducials including ease of injection, adaptable size, reduced imaging artefacts and negligible effects on radiation dose. ^16^^,^^21^^–^^23^ BioXmark ^®^ has the scope to increase precision and accuracy for the development of innovative preclinical RT studies. Increasing the precision of RT doses can reduce error in treatment targeting and thus reduce the need for large study numbers. In addition, reduced RT fields will prevent unnecessary dosing and RT toxicity to surrounding normal tissues, significantly improving animal welfare all within the framework of the NC3Rs. ^24^^–^^26^ BioXmark ^®^ has been successfully evaluated and deemed advantageous compared to other solid FMs for use in RT treatment in both clinical ^22^^,^^27^^–^^33^ and preclinical settings. ^34^^,^^35^

Successful refinement of radiotherapy dosing has the potential to impact over 500 mice required annually for radiotherapy studies within the Patrick G. Johnston Cancer Centre, upwards of 4,000 mice per year would benefit nationally, and over 30,000 mice per year internationally.

The purpose of this study is to demonstrate the versatility and ease of use of BioXmark ^®^ in preclinical applications. This article provides guidelines for the injection of BioXmark ^®^ in mouse models to aid visualisation of treatment targets and standardisation of treatment alignment for future preclinical RT treatment set-ups.

## Methods

### BioXmark ^®^

BioXmark ^®^ is a liquid FM produced by Nanovi A/S (Kongens Lyngby, Denmark). It is a sterile, ready-to-inject liquid composed of biodegradable sucrose acetate isobutyrate (SAIB), iodinated SAIB and ethanol. This formulation ensures that when BioXmark ^®^ is injected into soft tissue the ethanol partly diffuses out of the marker, increasing its viscosity, resulting in the formation of a semi-solid gel. ^36^ BioXmark ^®^ is visible on magnetic resonance imaging (MRI) and ultrasonography due to the SAIB component and visible on X-ray imaging modalities due to the electron-dense iodinated SAIB component. ^32^ Evidence of the visibility of BioXmark ^®^ on multiple imaging modalities in clinical, preclinical and phantom studies is presented in Table 1.

**Table 1.** Combination of clinical, preclinical and phantom studies published outputs stating the visibility of different volumes of BioXmark ^®^ on different imaging modalities.

	Injected volume of BioXmark ^®^						
Imaging Modality	5 μl	10 μl	15 μl	25 μl	40 μl	50 μl	100 μl
***Clinical***							
**CT** ^29^^,^^30^^,^^33^				Visible		Visible	Visible
**CBCT** ^27^^,^^29^^,^^30^^,^^33^				Visible		Visible	Visible
**MRI** ^29^^,^^30^				Visible		Visible	Visible with aid from corresponding CT scan
**Fluoroscopy** ^27^^,^^30^							Visible with aid from corresponding CT scan
***Preclinical***							
**CBCT (60kV) – SARRP** ^34^^,^^35^		Visible		Visible	Visible	Visible	
***Phantom & ex vivo models***							
**Planar 2D X-ray imaging** ^16^^,^^23^^,^^51^			Visible	Visible	Visible	Visible	Visible
**CT** ^16^^,^^23^^,^^51^	Visible	Visible	Visible	Visible	Visible	Visible	Visible
**CBCT** ^16^^,^^23^	Visible	Visible	Visible	Visible	Visible	Visible	Visible
**MRI** ^21^^,^^51^						Visible	Visible
**Preclinical CBCT (40, 50 & 60 kV) – SARRP** ^35^		Visible		Visible	Visible		
**Small-animal micro-CT (37 kV)** ^34^		Visible		Visible	Visible	Visible	

BioXmark ^®^ has been assessed over multiple imaging modalities by independent research centres and deemed visible from volumes as small as 5 μl preclinically and 25 μl clinically. Although visible on fluoroscopy and some MR linacs, confirmation is required from corresponding CT scans. Grey boxes indicate volumes for which there is no published data.

Like other FMs, it is recommended clinically to avoid the injection of BioXmark ^®^ into necrotic tissue, highly vascularised tumour tissue or air-filled cavities. ^36^ Preclinical injection of BioXmark ^®^ should also avoid these tissues and always be through the least invasive procedure possible and actively avoid the need for surgical implantation. Volumes over 50 μl are not recommended for use in mouse models due to negative imaging artefacts on preclinical CBCT scans. An optimum marker volume is suggested between 10-20 μl, this will allow visualisation on CBCT scans without hindering visualisation of small anatomical structures. ^35^ Larger volumes may be more applicable for clinical use or larger animal species.

### Animal models

All experimental procedures were carried out in accordance with the Home Office Guidance on the Operation of the Animals (Scientific Procedures) Act 1986, and approved by the Queen’s University Belfast Animal Welfare and Ethical Review Body (PPL2813). Animals were euthanized by Schedule 1 procedures. Animal studies are reported in compliance with the ARRIVE guidelines. ^37^

All mice were obtained from Charles River Laboratories (Oxford, UK). A mix of mice ages and genders were used due to animal availability from other ongoing studies. Female 12–15-week-old C57BL/6J mice were used for subcutaneous (n=3) and intra-peritoneal injections (n=2), male 14-18-week-old C57BL/6J mice were used for intra-tumoral study (n=21 BioXmark ^®^ injected tumours, n=21 control tumours) and male 10-15-week-old C57BL/6J mice were used for prostate injections (n=2). These numbers were used to assess the feasibility of injection types. No criteria were set to exclude animals from these studies. Mice were randomized to receive either a subcutaneous, or intra-peritoneal injections using an online random sequence generator. Blinding was not possible for intra-tumoral and prostate injections. Blinding was also not possible for image analysis as the injected FM, BioXmark ^®^, was clearly visualized on CBCT scans.

All mice were housed under controlled conditions (12-hour light–dark cycle, 21°C) in standard caging and received a standard laboratory diet and water *ad libitum.* To improve the welfare of mice environmental enrichment tools were placed in all cages including cardboard tubes for exploration, softwood blocks to encourage gnawing to prevent teeth overgrowth, nesting material for comfort and mouse swings for added cage complexity and exercise. Mice were also handled gently using refined cupping methods and frequently from a young age to reduce stress.

Mice were anaesthetised with injectable ketamine and xylazine (100 mg/kg and 10 mg/kg) prior to BioXmark ^®^ injection and CBCT imaging. All mice were placed in a heat-box (37°C) for recovery and monitored closely after injections until conscious and returned to normal behaviour.

### BioXmark ^®^ uptake

A 1 ml micro-dose syringe (Vlow Medical, Netherlands) was used for the uptake and injection of BioXmark ^®^. Micro-dose syringes are recommended as they ensure a controlled injection volume. A loading 18-gauge needle was used to fill the syringe slowly from the glass ampoule. BioXmark ^®^ is a viscous liquid so thinner needles may require a longer time to fill the syringe. Air bubbles were checked for through visual inspection prior to injection and were removed by gently tapping the side of the syringe and any excess or leakage BioXmark ^®^ was cleaned using ethanol wipes. The loading needle was removed after uptake into the syringe and replaced with a smaller needle (25-gauge) for each injection. Needles were replaced prior to each injection.

### Subcutaneous injection of BioXmark ^®^

BioXmark ^®^ was loaded into a micro-dose syringe as detailed above. For this study, we used a micro-dose syringe with options for the injection of 10, 20 and 40 μl. Mice were anaesthetised with injectable ketamine and xylazine (100 mg/kg and 10 mg/kg) before injection. A small area of fur on each flank was shaved and cleaned with an ethanol wipe for injection. 25-gauge needles were used for subcutaneous injections. The skin of the mouse was tented using the thumb and finger and a preselected volume of BioXmark ^®^ (10 μl (n=1), 20 μl (n=1) or 40 μl (n=1)) was injected under consistent pressure. Each mouse received two injections of BioXmark ^®^, one on each flank, e.g. 10 μl on the left and 10 μl on the right. As BioXmark ^®^ is very viscous the needle was held for 30 seconds after the volume was injected and then slowly removed. All mice were placed in a heat-box for recovery (37°C) and monitored closely until fully recovered from the anaesthetic and then returned to their original cage.

### Intraperitoneal injection of BioXmark ^®^

For this study, we used a micro-dose syringe (20 and 40 μl) and 25-gauge needles for injections (20 μl, n=1, 40 μl, n=1). Mice were anaesthetised with injectable ketamine and xylazine (100 mg/kg and 10 mg/kg) before the study, and the injection point sterilized with ethanol before intraperitoneal injection. As BioXmark ^®^ is very viscous, the needle was held for 30 seconds following injection and then slowly removed. No mice presented with bleeding or leakage of BioXmark ^®^, if there was leakage of BioXmark ^®^ this could be removed with an ethanol wipe. Mice were placed in a heat-box for recovery (37°C) and monitored closely until fully recovered from the anaesthetic before returning to their original cage.

### Intra-tumoral injection of BioXmark ^®^

A previous study reported that it was not possible to mix BioXmark ^®^ with tumour cells and a solid tumour must be established prior to injection of BioXmark ^®^. ^34^ In this study, we trialled an intra-tumoral injection of BioXmark ^®^ as outlined in Brown *et al.* ^35^

Tumour xenograft studies were performed using MC38 colon cancer cells (originate from James W. Hodge) cultured in DMEM media supplemented with 10% foetal bovine serum (FBS) and 1% penicillin/streptomycin. Cells were maintained at 37°C in a humidified atmosphere of 5% CO _2_ and subcultured every 3–4 days to maintain exponential growth.

MC38 cells were cultured *in vitro* and prepared in PBS (1 × 10 ^5^ cells per 100 μl). Subsequently, 100 μl was injected subcutaneously into the flank of each C57BL/6J mouse (n=42). Mice were anesthetized using inhalant isoflurane (0.5 – 2%) for implant and placed in a heat box for recovery. Mice were then returned to conventional housing and closely monitored. Tumour volume was determined three times a week using calliper measurements in three orthogonal dimensions. Once tumours grew to a volume of 100 mm ^3^ mice were randomised using an online random sequence generator into control (n=21) and BioXmark ^®^ (n=21) cohorts. This injection method was used to assess the radiobiological effect of BioXmark ^®^ with tumours treated with single (16 Gy) or fractionated (2×8 Gy or 3×4 Gy) doses of RT. These mice were treated with RT as outlined in Brown *et al.* ^35^

For the intra-tumoral injection of BioXmark ^®^, the FM was loaded into a micro-syringe as detailed above and 25-gauge needles were used for injection. Mice were anaesthetised with injectable ketamine and xylazine (100 mg/kg and 10 mg/kg) and the injection point sterilized with ethanol before injection. The needle was carefully placed into the middle of the tumour (estimated through needle insertion) and 20 μl of BioXmark ^®^ injected under consistent pressure. To prevent leakage or bleeding the needle was held for 30 seconds before removal. No mice presented with bleeding or leakage of BioXmark ^®^. All mice were placed in a heat-box for recovery (37°C) and monitored closely until fully recovered from the anaesthetic.

### Orthotopic prostate injection of BioXmark ^®^

Intra-prostate injections of BioXmark ^®^ were performed under aseptic conditions (n=2). Mice were anaesthetised using inhalant isoflurane (0.5–2%) throughout the procedure and administered analgesia (buprenorphine 0.015 mg/ml (0.05 mg/kg dose)) via IP injection before the surgical procedure and 6 hours post. Protocol adapted from Pavese *et al.* ^38^ A small portion skin was shaved and the prostate exposed through a low midline abdominal incision of 5 mm (roughly 1 cm above external genitals). The anterior lobe was identified, and a 26-gauge needle was used to inject 10 μl of BioXmark ^®^ under consistent pressure and the needle held in place for 30 seconds to prevent leakage. Due to the delicate procedure, we trialled a finer gauge needle and found it feasible to inject BioXmark ^®^ effectively with a 26-gauge needle. Successful injection was confirmed by the formation of a small rounding or mound shape. The injection area was checked for bleeding or leakage of BioXmark ^®^, none was found to leak, before carefully placing the prostate back into the abdominal cavity and the small wound was sealed with tissue glue and stitches. Mice were placed in a heat box for recovery (37°C) and healing, body weight and behaviour were closely monitored after the surgical procedure and then returned to original caging.

### Injection protocols

**BioXmark ^®^ uptake**

A. BioXmark ^®^ should be stored at room temperature in a sealed glass ampoule.
B. Prepare the syringe (micro-dose injector, vlow medical 1ml) by adding a loading needle (18-gauge).
C. Carefully open the BioXmark ^®^ ampoule and fill the syringe with the transparent liquid using the loading needle.
  - BioXmark ^®^ is a viscous liquid so thinner needles may require a longer time to fill the syringe.
  - Remove bubbles by gently tapping the syringe.
  - Clean excess or spilled BioXmark ^®^ with an ethanol wipe.
  - BioXmark ^®^ is single use, dispose of excess when finished.
D. Once all the liquid has been taken up into the syringe change the loading needle to a new needle for injection (25-gauge or 26-gauge).
E. Fill the new needle with BioXmark ^®^ liquid ensuring no air bubbles are present.
F. Clean any excess BioXmark ^®^ from the needle with an ethanol wipe before injection.

**Injection of BioXmark ^®^**

A. Place anaesthetised mouse on a clean surface and shave a small area of fur (if required) on the flank of mouse at the point of implant.
  - Once ready for implant suitably restrain, depending on injection route, the mouse for injection.
B. Use an ethanol wipe to clean the skin.
C. Insert the 25-gauge/26-gauge needle (bevel side down) and inject the volume of BioXmark ^®^ required under consistent pressure. Hold for 30 seconds before slowly removing the needle.
D. Check area for bleeding or leakage of BioXmark ^®^ and carefully clean with ethanol wipe if needed.
  - If bleeding occurs, check the source of bleeding to ensure no internal organs have been punctured or pain caused to the animal. Apply pressure with clean gauze. If bleeding does not stop take appropriate measure e.g. additional monitoring, pain relief, remove from experiment.
E. If multiple injections of BioXmark ^®^ are required, replace needle and repeat injection process.
F. Place mouse in a heat-box for recovery (37°C) and monitor closely until fully recovered from anaesthetic.

**Intra-tumoral injection of BioXmark ^®^**

**Figure 1.**
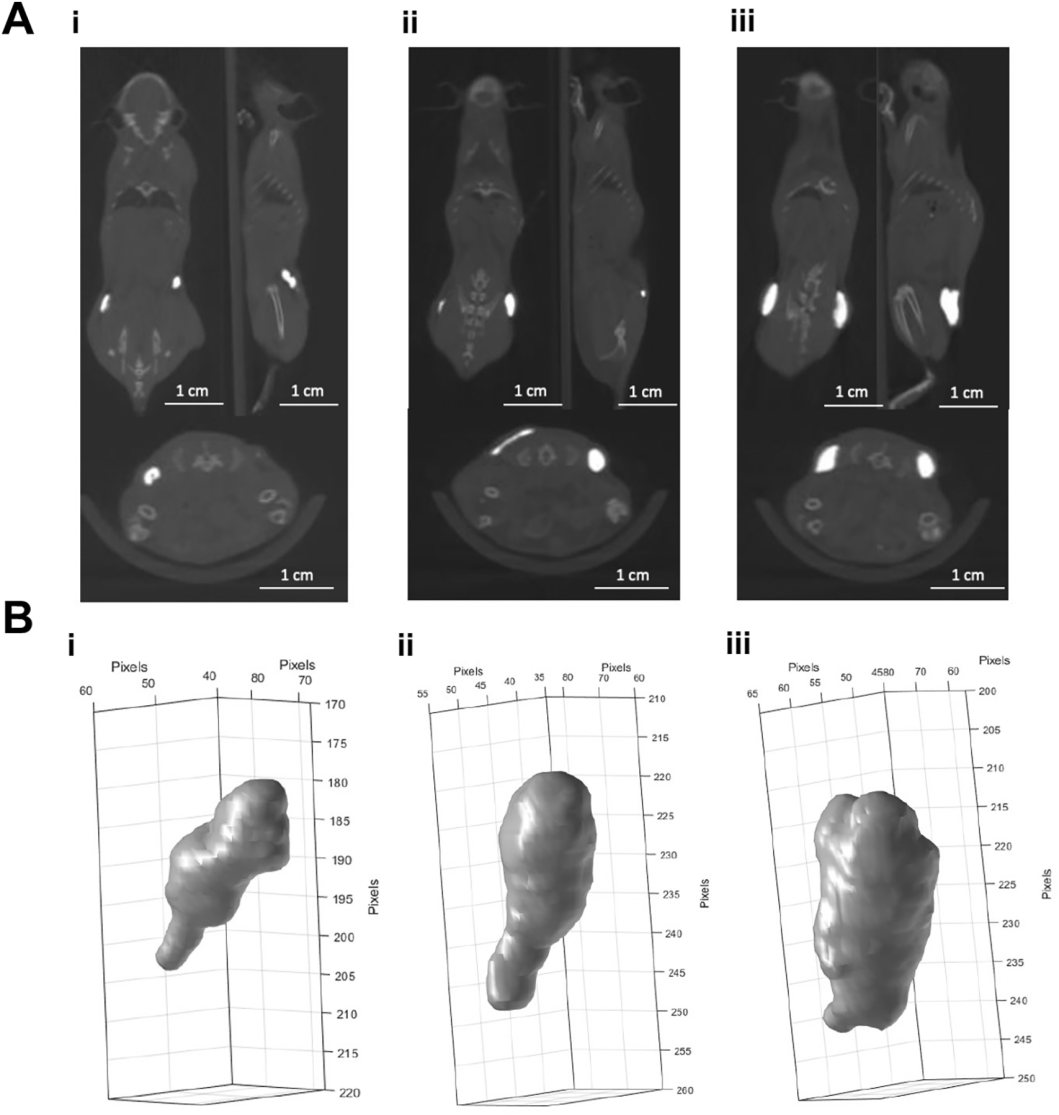
*In vivo* CBCT imaging analysis of the different volumes of BioXmark ^®^ following subcutaneous injection. Panel A: CBCT scans of 10 (i), 20 (ii) and 40 µl (iii) of BioXmark ^®^ (left to right) injected subcutaneously at two points on the flank of mice (n =1). Panel B: Volumes of BioXmark ^®^ on CBCT scans were contoured using CERR software and 3-D shape of each marker visualized.

A previous study trialled mixing BioXmark ^®^ with tumour cells prior to implant for targeting, this was not feasible with the tumour needing to be establish prior to BioXmark ^®^ injection. ^34^ In this study we trialled an intra-tumoral injection of BioXmark ^®^ as outlined in Brown *et al* 2020. ^35^ Mice were randomised into control of BioXmark ^®^ injected cohorts once sucutaneous tumours reached a suitable size for treatment (100 mm ^3^). This protocol was used to determine the radiobiological effect of BioXmark ^®^ on tumour response, cytotoxicity and dose perturbation. ^35^ For tumour targeting multiple subcutaneous injections of BioXmark ^®^ around the tumour would be more suitable ( Figure 1).

A. Ensure needle and syringe are loaded with BioXmark ^®^ and are prepared for injection.
B. Place anaesthetised mouse on a clean surface and use an ethanol wipe to sterilise the point of injection.
C. Insert the 25-gauge/26-gauge needle (bevel side down) to the middle of the tumour.
D. Inject the desired volume of BioXmark ^®^ (10-25 μl) under consistent pressure and wait 30 seconds before removing the needle.
E. Check area for bleeding or leakage of BioXmark ^®^ and carefully clean with ethanol wipe if needed.
  - If bleeding occurs, check source and apply pressure to stop. Take appropriate measures if needed e.g. additional monitoring.
F. Place mouse in a heat-box for recovery (37°C) and monitor closely until fully recovered from anaesthetic.

**Orthotopic prostate injection of BioXmark ^®^**

Mice were anaesthetised using inhalant isoflurane throughout the procedure and administered analgesia (buprenorphine 0.015 mg/ml (0.05 mg/kg dose)) prior to surgical procedure and 6 hours post. Protocol adapted from Pavese *et al* 2013. ^39^

A. Procedure to be performed under aseptic conditions.
B. Ensure needle and syringe are loaded with BioXmark ^®^ and are prepared for injection.
C. Expose the prostate through a low midline abdominal incision of 5 mm (roughly 1 cm above external genitals).
D. Once the prostate is exposed identify the lobes.
E. Using a 26-gauge needle, inject 10 μl of BioXmark ^®^ into the anterior lobe of the prostate under consistent pressure.
F. To ensure successful injection, check a small bubble had formed. Hold the needle in place for 30 seconds before removal to prevent leakage.
G. Check area for bleeding or leakage of BioXmark ^®^ and carefully clean with cotton tip if needed.
  - If bleeding occurs, check source of bleed to ensure no internal organs have been punctured or pain caused to the animal. Use sterile cotton tip to clean the area/apply pressure. Take appropriate measures if needed e.g. additional monitoring, pain relief, remove from experiment.
H. Carefully place the prostate back into the abdominal cavity.
I. Use tissue glue and stiches to close the small wound.
J. Place mouse in a heat box for recovery (37°C).
  - Or suitable equivalent e.g. cage placed on heat mat
K. Closely monitor mouse after surgical procedure.
  - In particular wound healing, body weight and behaviour.

### Preclinical imaging

Imaging was completed using the Small Animal Radiation Research Platform (SARRP) (Xstrahl Life Sciences, Camberley UK) onboard CBCT imaging. An imaging energy of 60 kV was used with 0.5 mm Al filtration for all *in vivo* models. The acquired CBCT scans were transformed into material properties by defining five discrete windows for these materials; air, lung, fat, tissue and bone. ^39^ Imaging was completed immediately after the injection of BioXmark ^®^, while the mice were still anaesthetised, for subcutaneous, IP and intra-tumoral injections. Mice injected with BioXmark ^®^ into the prostate were allowed to recover overnight in the home cage and imaged the following day. These mice were anaesthetised with injectable ketamine and xylazine (100 mg/kg and 10 mg/kg) for imaging.

### CERR analysis

Computational Environment for Radiological Research (CERR) software ( https://cerr.github.io/CERR/) was used to complete additional image analysis. Image analysis could not be blinded as BioXmark ^®^ was visible on the CBCT scans. For contouring of BioXmark ^®^, all relevant DICOM images and structure sets were exported into CERR software within MATLAB (Version 2019b). ^40^ Structures of interest were contoured manually using the CERR in-house contouring interface. Contours were created slice-by-slice in the coronal plane with corrections made using the sagittal and axial planes. To reduce variations in contours all structures were contoured by the same user.

### Statistical analysis

Statistical differences were calculated using unpaired two-tailed student t-tests, or one-way ANOVA tests where appropriate, with a significance threshold of p < 0.05 using Prism GraphPad Prism 7 (Version 7.01, GraphPad Software, Inc.). Data is presented either as the average for the entire experimental arm ± standard error (SEM).

## Results

### *In vivo* injection techniques

This study has provided methods for the application of BioXmark ^®^ in small animal models. Mice of both male and female sexes were used and no behavioural or toxic side-effects due to BioXmark ^®^ were observed.

Figure 1A shows CBCT scans from mice injected subcutaneously with BioXmark ^®^ at two separate points on the flank (10, 20 or 40 μl). BioXmark ^®^ is easily visualised in bright white on all CBCT scans and could be easily differentiated from anatomical structures ( Figure 1A). Capsules of BioXmark ^®^ formed in the cavity under the skin with all volumes having a similar 3-D shape ( Figure 1B & Supplementary Figure 1). Further analysis of the long-term (5-month) stability of BioXmark ^®^ injected subcutaneously is detailed in Brown *et al.* ^35^

**Figure 2.**
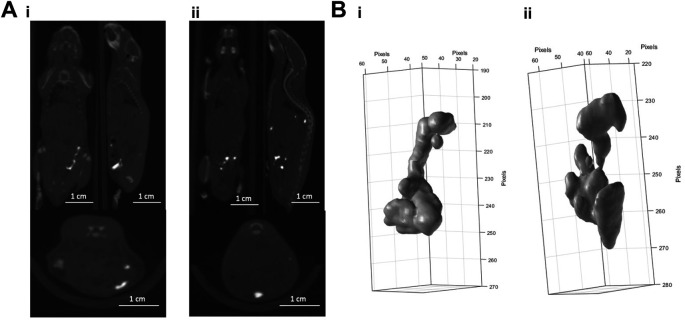
*In vivo* CBCT imaging analysis of the intraperitoneal injection of different volumes of BioXmark ^®^. Panel A: CBCT scans of 20 µl (i) and 40 µl (ii) of BioXmark ^®^ injected via intraperitoneal injection (n=1 per dose). Panel B: 3-D visualisation of 20 µl (i) and 40 µl (ii) of BioXmark ^®^ after intraperitoneal injection.

Due to differences in marker shape between subcutaneous ( Figure 1A) and intraperitoneal injections ( Figure 2A) we assume that BioXmark ^®^ moulds around anatomical structures before solidifying. There is greater diversity in the shape after injection into the intraperitoneal cavity with the most evidenced by the larger volume of 40 μl ( Figure 2A & B), indicating that the intraperitoneal injection is less stable than other methods with the marker breaking up and potentially solidifying in a different area than injected.

**Figure 3.**
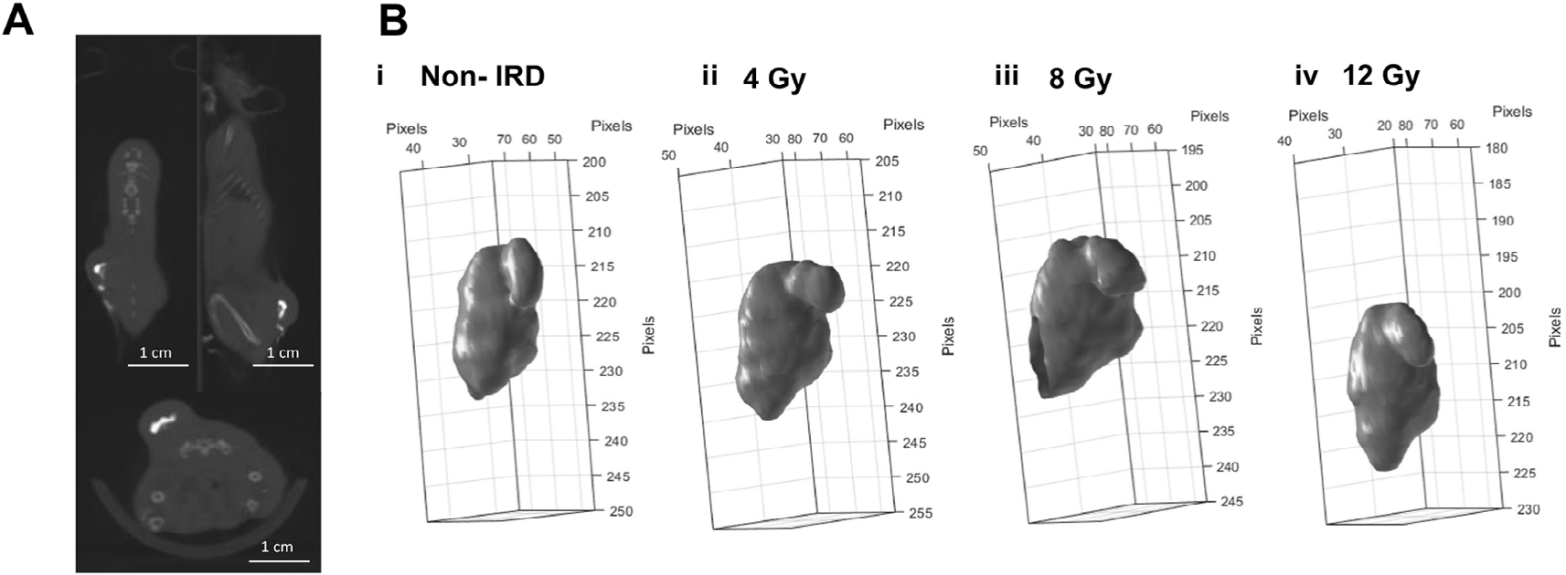
*In vivo* CBCT imaging analysis of the intra-tumoral injection of 20 µl of BioXmark ^®^. 20 µl was injected into MC38 subcutaneous tumours once they reached a volume of 100 mm ^3^. Panel A: Mice were imaged on CBCT at 60 kV prior to receiving radiotherapy (n=21). Panel B: Volumes of BioXmark ^®^ on CBCT scans were contoured using CERR software and the 3-D shape of each marker was visualised throughout the dose schedule.

Tumour volumes have different shapes and sizes and are heterogeneous causing differences in diffusion of a substance, such as drugs or contrast agents, and potentially BioXmark ^®^. Figure 3A shows the injection of 20 μl of BioXmark ^®^ into a subcutaneous MC38 colon carcinoma tumour. This was completed to trial the feasibility of injecting BioXmark ^®^ into dense tissue. Intra-tumoral injections were visualised as a hyperdense structure in the centre of the tumour easily differentiated from the tumour tissue ( Figure 3A). 3-D reconstructions of injected BioXmark ^®^ which underwent fractionated RT (3×4 Gy) are shown in Figure 3B. Minimal changes were observed in the shape of BioXmark ^®^. No radiobiological effects of BioXmark ^®^ were determined, further detailed in Brown *et al.* ^35^

**Figure 4.**
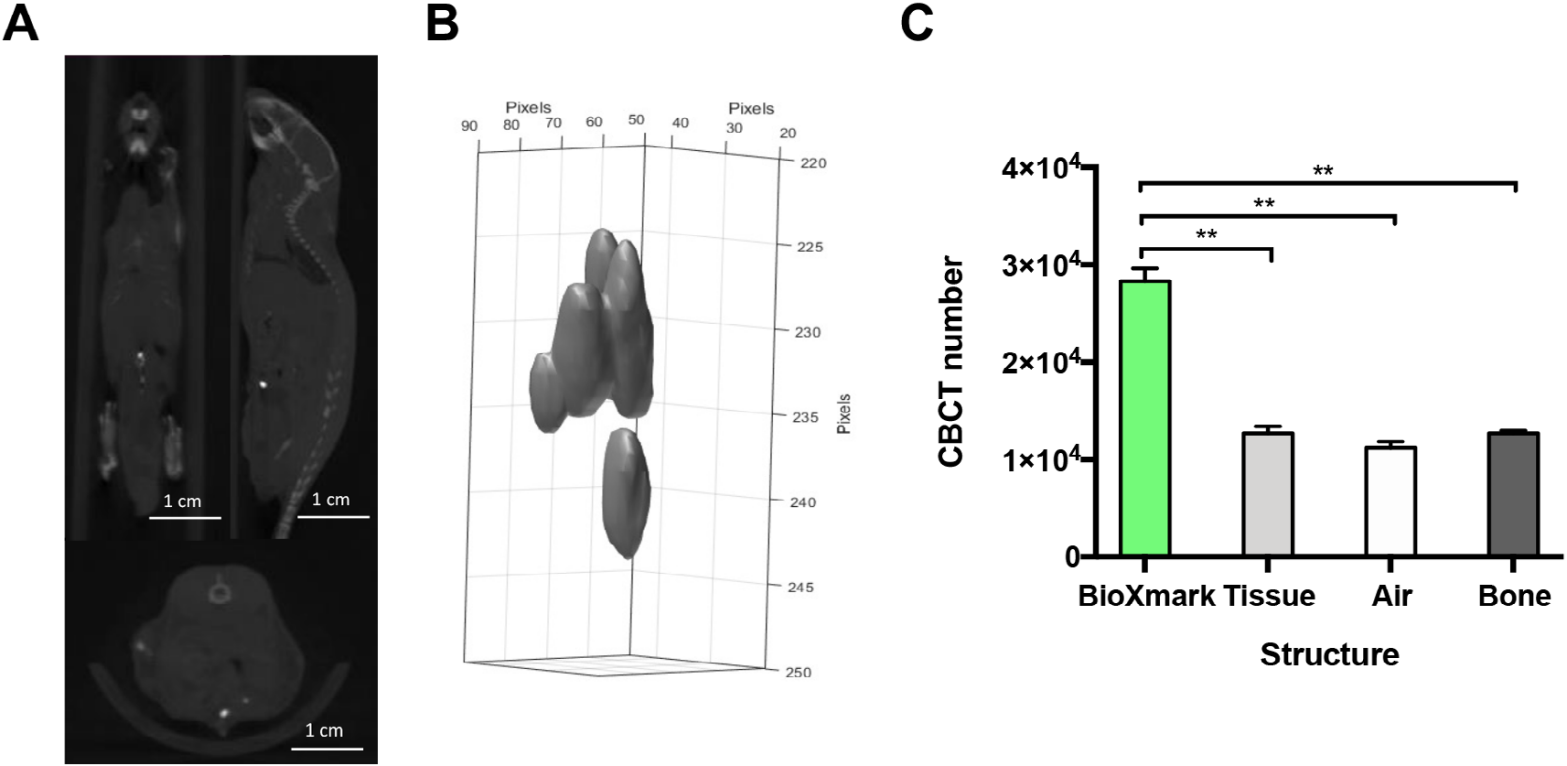
*In vivo* CBCT imaging analysis of the prostate orthotopic injection of 10 µl of BioXmark ^®^. Panel A: Mice were imaged on CBCT at 60 kV after injection of BioXmark ^®^ (n=2). Panel B: 3-D shape of injected BioXmark ^®^. Panel C: Viability of the marker was quantified through CBCT numbers for BioXmark ^®^, tissue, air and bone. Data presented are mean values ± SEM; statistical significance is reported as *< 0.05, ** < 0.01.

The injection of 10 μl of BioXmark ^®^ into an orthotopic prostate model is shown in Figure 4A. This method is the most technical and clinically accurate for placement of a FM for RT treatment targeting. The orientation of the marker can be monitored through 3-D analysis providing additional information on the location of the prostate. BioXmark ^®^ can be clearly visualised from CBCT scans but also differentiated from soft tissue through CBCT values for tissue ( *p*=0.0093), air ( *p*=0.0072) and bone ( *p*=0.0073) ( Figure 4C). Injected BioXmark ^®^ can help to identify the prostate to improve defining a target volume for beam delivery. This model could be used for normal tissue targeting of the prostate or for targeting orthotopic prostate tumours.

## Discussion

This study is the first to provide a detailed methodology for the use of the liquid FM BioXmark ^®^ in preclinical mouse models. The feasibility of multiple injection methods has been trialled for use of the marker as a reference point for submillimetre targeting and beam positioning in preclinical RT set-ups. In this study, we assessed the visibility and 3-D profile of BioXmark ^®^ for subcutaneous, intraperitoneal, intra-tumoral and orthotopic prostate injections. All injection types were feasible and easily performed with BioXmark ^®^ easily differentiated from anatomical structures on CBCT scans. However, the IP injection of BioXmark ^®^ would not be recommended for use in RT targeting due to movement through the IP cavity, it may be more applicable to use a contrast agent for IP injections. We would recommend injection volumes between 10-20 μl of BioXmark ^®^ for use in mouse models. This study complements previous experimental studies for the transferability of BioXmark ^®^ between laboratories to improve treatment targeting in preclinical models of RT. ^34^^,^^41^

When BioXmark ^®^ is injected into soft tissue it forms a semi-solid gel, this acts as an anchor for the FM. Stability is an essential property of FMs for treatment targeting. ^14^ Movement or loss of a marker can lead to inaccurate dose deposition and reduces the efficiency of RT treatment which may be increasingly important during hypo-fractionated schedules. ^42^ Other liquid FMs are not suitable for fractionated RT as they have been reported to not solidify after injection (Lipiodol) or are not stable for long periods (hydrogel). ^16^^,^^43^ The iodine-containing contrast agent Imeron 300 has been used preclinically for contrast-enhanced CBCT scans. Dobiasch *et al* have compared Imeron 300 with BioXmark ^®^ for targeting pancreatic tumours in mice; whilst both were easily visualised on CBCT scans BioXmark ^®^ was concluded more suitable for targeted RT. ^34^ Imeron 300 has to be continually administered for scanning, adding additional stress on experimental mice. ^44^ BioXmark ^®^ biodegrades slowly with stability shown up to 6 months preclinically. ^35^ De Blanck *et al* estimate that full marker degradation is up to 3 years clinically ^31^; therefore, BioXmark ^®^ is ideal for outlining treatment parameters for fractionated RT and treatment follow-up.

As a liquid FM, BioXmark ^®^ has advantages over solid FMs with the volume more controllable making it adaptable for preclinical use. The volume of the marker can be target specific and as small as 5-10 μl ( Table 1). ^34^^,^^35^ Once injected these markers conform to complex shapes surrounding soft tissue or tumour borders for 3-D visualisation. The final shape of injected BioXmark ^®^ can provide crucial information on the shape, size and rotation of a tumour which cannot be achieved with solid FMs. ^16^^,^^23^^,^^45^ Any changes to the shape of BioXmark ^®^ after irradiation have yet to be reported from current clinical and preclinical studies. We were able to monitor the 3-D shape of intratumorally injected BioXmark ^®^ throughout fractionated treatments ( Figure 3B) and showed that the 3-D shape was not significantly affected by 4 Gy fractions of RT. Small changes observed were expected due to changes in tumour shape after irradiation. However, we would recommend using peri-tumoral injections i.e. at multiple points surrounding or adjacent to a tumour, of BioXmark ^®^ for future studies to better align with clinical set-ups and remove BioXmark ^®^ from the treatment volume.

FMs or fixed contrast agents are essential to improve the targeting of tumours in the lower abdomen and prostate in small animal RT. Current set-ups have high levels of errors due to large target fields which reduce the clinical relevance of studies and cause high levels of normal tissue toxicity. ^44^^,^^46^^,^^47^ In addition, there are significant targeting errors in fractionated treatment schedules due to a lack of stable reference points. Verginadis *et al* used a solid radio-opaque marker for implantation to the jejunum of a mouse to improve targeting. This study reported increased toxicity levels and blockage of the GI tract. ^48^ BioXmark ^®^ is an alternative marker with no reports of toxic side effects preclinically. ^34^^,^^35^ We have demonstrated the ability to use BioXmark ^®^ for the targeting of prostate tumours with clear differentiation of BioXmark ^®^ from anatomical structures ( Figure 4C). This approach can be adapted to other orthotopic tumour models to improve the reproducibility of RT targeting and significantly reduce the risk of adverse effects in mice.

## Conclusion

Despite advances in preclinical research, only one-third of *in vivo* studies translate to clinical trials. ^49^ This is largely due to preclinical studies not replicating the clinical setting, and a lack of comprehensive reporting of methods, data sharing and transferability of protocols between laboratories. ^50^ This study shows the feasibility of using BioXmark ^®^ in preclinical models of RT, it’s use can be adapted within preclinical radiobiology centres worldwide to improve the visualisation of orthotopic tumours and the translational impact of studies. Standardising the coupling of preclinical RT platforms with targeted imaging tools such as BioXmark ^®^ is a huge step toward quality assurance, reducing targeting uncertainties and most importantly improving animal welfare.

## Author contributions

Conceptualization, K.T.B., K.M.P.; Methodology, data curation and investigation, K.H.B., M.G., K.T.B.; Writing – Original Draft Preparation, K.H.B., K.T.B.; Writing- review & editing, M.G., K.M.P. All authors have read and agreed to the published version of the manuscript.

## Data Availability

Figshare. Preclinical CBCT scans. DOI: https://doi.org/10.6084/m9.figshare.22227490.v1 ^52^ This project contains the following data:
- CBCT datasets to accompany “Feasibility and guidelines for the use of an injectable fiducial marker (BioXmark ^®^) to improve target delineation in preclinical radiotherapy studies using mouse models.” Data are available under the terms of the Creative Commons Attribution 4.0 International license (CC-BY 4.0). Figshare. ARRIVE checklist. DOI: https://doi.org/10.6084/m9.figshare.22227478.v1
- ARRIVE checklist to accompany “Feasibility and guidelines for the use of an injectable fiducial marker (BioXmark ^®^) to improve target delineation in preclinical radiotherapy studies using mouse models.” Data are available under the terms of the Creative Commons Zero “No rights reserved” data waiver (CC0 1.0 Public domain dedication).
