# Supplementary material for: Feasibility and guidelines for the use of an injectable fiducial marker (BioXmark ®) to improve target delineation in preclinical radiotherapy studies using mouse models
Source: F1000Res. 2023 May 22;12:526. [Version 1] doi: 10.12688/f1000research.130883.1 (PMC11116939; doi:10.12688/f1000research.130883.1)
Supplement: Peer review history

# Reviewer report: Reviewer response for version 1

**DOI:** 10.5256/f1000research.143675.r274478

**Published:** 2024-05-23

**Authors:** Katrina Stevenson

**Affiliations:** 1 School of Cancer Sciences, University of Glasgow, Glasgow, Scotland, UK

In this paper the researchers aim to demonstrate the versatility and ease of use of BioXmark ^®^ in preclinical applications.

 This study provides a good comprehensive technical overview of how BioXmark ^®^ works and how to use it into your pre-clinical imaging studies. The details of the protocols used would allow the FM to be easily incorporated into the practice in other establishments.

 This research shows that BioXmark ^®^ would be useful in preclinical imaging to delineate the area of tumour which would improve the accuracy of dosing mice with radiation in small animal radiation systems therefore reducing normal tissue irradiation toxicity. This aligns with the use of fiducial markers in clinical practice further validating the use in preclinical imaging and radiation.

 The study highlights the impact that using BioXmark ^®^ could make on the number of mice used in imaging and radiations studies – cutting down required amount as you are more assured of the accuracy and eliminating poorly targeted tumours. Thus, making the method interesting in terms of the NC3Rs framework.

 Methods set out how the study used BioXmark ^®^ in all injection methodologies discussed – intra-tumoral, intraperitoneal, subcutaneous and orthotopic. Detailed instruction on handling and injecting the reagents are given. Numbers of mice used is given - some experimental numbers used are low but as this is a proof of principle, technical study, are adequate. Both males and females were used, and no toxicity was seen.

 Results give a good indication of how the FM would look in preclinical models with examples for each methodology – ruling out the use of intraperitoneal injection due to the disruption in stability of BioXmark ^®^. They have also shown the volumes of the FM using the CERR software and this shows a 3-D visualisation of the FM. Placement of the BioXmark ^®^ in the orthotopic prostate model proved the most technically challenging but would give good accuracy in dosing the prostate – helping to prove clinical relevance of using this FM in preclinical studies. Long term stability of BioXmark ^®^ has been previously discussed in Brown et. al., reference 35.

 Discussion clearly describes how this study has shown the benefits of using this liquid FM in preclinical studies as an aide to increasing targeting accuracy.

 An interesting area of study not covered in this research would be to look at how the BioXmark ^®^ interferes with the dose given to the to the tissue – if there was a differential in dose calculated by the preclinical imaging and radiation software – this had been touched on a little in the referenced previous study by Brown et. al., reference 35.

 It would have been good to have details of the scheduling of the fractionated doses of radiation in the methods/results sections.

 Methods/protocols sections - the text is quite repetitive.

Are the 3Rs implications of the work described accurately?

Yes

Is the work clearly and accurately presented and does it cite the current literature?

Yes

If applicable, is the statistical analysis and its interpretation appropriate?

Yes

Are all the source data underlying the results available to ensure full reproducibility?

Yes

Is the study design appropriate and is the work technically sound?

Yes

Are the conclusions drawn adequately supported by the results?

Yes

Are a suitable application and appropriate end-users identified?

Yes

Are sufficient details of methods and analysis provided to allow replication by others?

Yes

Reviewer Expertise:

Preclinical radiation studies/preclinical cancer models

I confirm that I have read this submission and believe that I have an appropriate level of expertise to confirm that it is of an acceptable scientific standard.

# Reviewer report: Reviewer response for version 1

**DOI:** 10.5256/f1000research.143675.r215285

**Published:** 2024-05-10

**Authors:** Jane Sosabowski, Rebecca Carter

**Affiliations:** 1 Barts Cancer Institute, Queen Mary University of London, London, UK; 2 Department of Medical Physics & Biomedical Engineering, University College London (Ringgold ID: 4919), London, England, UK

Title: The title of this manuscript summarises the content well: Mainly, the paper concerns development of injection protocols for a liquid fiducial marker (BioXmark) that can be used preclinically during CT imaging and radiotherapy.

Abstract:. please correct:..... to ensure accuracy **of** beam targeting. (new sentence)  This approach .....

Introduction: The Introduction does a good job of setting the scene for this study, outlining use in relation to clinical applications, describing the problems inherent in using solid FMs preclinically, and relating the study to improvements in animal welfare. However, in places, particularly paragraph 3, it is difficult to read, due to sub-optimal grammar or punctuation. This would be improved by re-reading and restructuring some of the longer sentences.

Methods: In general, the detail in the Methods section is excellent. The only issue is in the section on intra-tumoral injection, which states: ‘This protocol was used to determine the radiobiological effect of BioXmark® on tumour response, cytotoxicity and dose perturbation (35). Reference 35 (Brown et al 2020), is frequently referred to throughout the text, but relates to a different tumour cell line, Lewis Lung, rather than the MC38 cell line described here. To me, there is insufficient data on how these two studies are related. It would be helpful to add a bit more information and clarification on the two studies, if appropriate, to assist the reader.   

 Preclinical imaging method section:  "An imaging energy of 60 kV was used" - keV is not a unit of energy

Results: The Results presented are quite straightforward; firstly a characterization of BioXmark injection at subcutaneous (n=3), intra-peritoneal (n=2)and intra-prostate (n=2) sites; with methodological recommendations proposed for each location. The main contents of the Results show the appearance of the marker in whole mouse CT scans and a contour-derived representation of BioXmark distribution from within each tissue (Figures 1, 2 and 4). These sections provide useful information for other users, but did feel a bit limited. I would prefer that there was a summary of the effects of BioXmark on dose calculation at these locations, and if possible some information on the effect on measured radiation dose, or a reference to these, if indicated in an additional publication. It would be helpful to have confirmation that the Muriplan software can calculate accurate doses in regions containing the BioXmark, and that if this is an issue, how best it could be mitigated.

 However, this notwithstanding, the study provides a lot of detail on injection protocols that will be useful for researchers looking to use Bioxmark as an FM in pre-clinical irradiations. It is also informative as it shows the relative utility of BioXmark for injection at different sites.

 The remaining Results section, referring to Figure 3, details intra-tumoral injection (n=42) to demonstrate ‘the feasibility of injecting BioXmark® into dense tissue.’ and shows CT scans from one mouse and a representation of BioXmark distribution over the course of a 4x 4 Gy irradiation schedule, to highlight the minimal changes to BioXmark following irradiation. For this section, the timescale over which the 4x4 Gy fractions were delivered should be added. Also, there is a statement that ‘no radiobiological effects of the BioXmark were determined, further detailed in reference 35.’ However, reference 35 presents very limited detail on radiobiological effect, only dose calculation discrepancies and the impact that these may have on tumour growth following treatment. Therefore it might be best to restate in this current paper, the potential impact on dose calculation of BioXmark in the target volume. If there are any biological markers of radiation effect that have been analysed, in the presence/absence of BioXmark, following treatment, it would be good to mention that here.  If there is n=42 data, these should be presented.

Discussion and Conclusion: These were very clear and concise

Additional issues

 The text is a bit repetitive in places, also one section occurs twice, headed: ‘Intra-tumoral injection of BioXmark’ , and the first two sentences starting: ‘A previous study trialled mixing BioXmark® with tumour cells prior to implant for targeting…’  Therefore, one of the sections should be deleted.

 Statistical analysis is not particularly necessary but is provided to show the difference in CBCT values for radiodensity between the BioXmark and other tissues on the CT scan. In my opinion, it is not necessary to give exact p-values to multiple decimal places for these p-values, as large numbers of decimal places are harder to read. The test used to generate the p-values should be indicated. Please adjust this if possible.

Are the 3Rs implications of the work described accurately?

Yes

Is the work clearly and accurately presented and does it cite the current literature?

Yes

If applicable, is the statistical analysis and its interpretation appropriate?

Partly

Are all the source data underlying the results available to ensure full reproducibility?

Yes

Is the study design appropriate and is the work technically sound?

Yes

Are the conclusions drawn adequately supported by the results?

Yes

Are a suitable application and appropriate end-users identified?

Yes

Are sufficient details of methods and analysis provided to allow replication by others?

Yes

Reviewer Expertise:

Preclinical radiotherapy, preclinical imaging

We confirm that we have read this submission and believe that we have an appropriate level of expertise to confirm that it is of an acceptable scientific standard, however we have significant reservations, as outlined above.

# Reviewer report: Reviewer response for version 1

**DOI:** 10.5256/f1000research.143675.r215290

**Published:** 2023-11-09

**Authors:** Emma Biglin

**Affiliations:** 1 Division of Cancer Sciences, Faculty of Biology, Medicine and Health, The University of Manchester, Manchester, UK

The study by Brown *et al.* provides detailed guidelines for the use of the liquid fiducial marker (FM) BioXmark to help identify areas of interest within a cone-beam CT scan, with the overall aim of improving the targeting of radiation. To demonstrate the use of BioXmark, the authors tested the FM at four different injection sites. Capsules of BioXmark were easily identifiable at all injection sites, even in high density areas, however in the intraperitoneal site the FM broke down and migrated around the structure. BioXmark allows the identification of tumour sites for improved RT targeting, without impacting the image quality or inducing toxic side effects as seen with other FMs.

 Improved RT targeting will refine preclinical research by minimising potential toxicity to normal tissue and associated short- and long-term side effects. Increasing confidence in the dose delivered to the target also has the potential to reduce the numbers of animals required.

 The article is well written and provides an easily reproducible protocol to allow this technique to be adopted into routine protocol at other institutions. Use of this technique has a huge impact on preclinical RT research to both improve the reliability of results and animal welfare. I have a few minor comments that may improve the manuscript.

**Introduction**

1. The authors include a good introduction of the use of FMs, with the limitations of using solid FMs and how liquid FMs are more suitable in the RT context. Are there any limitations to using liquid FMs?

**Methods**

1. Table 1 is a good summary of the current uses of BioXmark but it is quite difficult to distinguish the subheadings within the table as they blend in with the other rows.
2. Paragraph 2 line 1 – can the authors describe why it is not recommended to inject BioXmark into necrotic tissue, highly vascularised tissue or air cavities and is there a way to avoid this?
3. In accordance with the ARRIVE guidelines can you include:
  i) The total number of mice used for the study – how many of these were from other studies?
  ii) How were the sample sizes calculated?
  iii)Were there any animals/data points not included in the analysis?
4. The authors describe the optimal volume to be 10-20ul, was there a particular reason why 10ul wasn’t used for the intraperitoneal site and 40ul was used for the subcutaneous and intraperitoneal sites?
5. Can the authors include the suppliers of all consumables: needles, cell culture reagents etc?
6. What radiobiological effects were assessed in the intra-tumoral group?
7. For the fractionated group, could you please clarify if these were on consecutive days?
8. “Injection of BioXmark protocol” – Is this for the subcutaneous and intraperitoneal injection sites?
9. Preclinical imaging – what software was used to define the material properties? Have you used the right reference here?

**Results**

1. Figure 3B – can you clarify that the different doses in the figure are accumulations of the individual fractions.
2. I would suggest adding details of the volume contouring to figure captions 2 and 4, to be consistent with figure captions 1 and 3.
3. Can the last two sentences of the final paragraph be combined as there is slight repetition? Would it sound better as “normal tissue sparing of the prostate” as it currently may sound like you are looking to target the prostate with radiation rather than identify it to make sure it is out of the radiation field?

**Discussion**

1. Paragraph 2, sentence 3 – to avoid confusion would it be better to say “movement or loss of a marker can lead to inaccurate targeting” rather than dose deposition as this reads like the FM deposits the dose.

Are the 3Rs implications of the work described accurately?

Yes

Is the work clearly and accurately presented and does it cite the current literature?

Yes

If applicable, is the statistical analysis and its interpretation appropriate?

Yes

Are all the source data underlying the results available to ensure full reproducibility?

Yes

Is the study design appropriate and is the work technically sound?

Yes

Are the conclusions drawn adequately supported by the results?

Yes

Are a suitable application and appropriate end-users identified?

Yes

Are sufficient details of methods and analysis provided to allow replication by others?

Yes

Reviewer Expertise:

Radiobiology, preclinical dosimetry

I confirm that I have read this submission and believe that I have an appropriate level of expertise to confirm that it is of an acceptable scientific standard, however I have significant reservations, as outlined above.
